# Giant cervical uterine leiomyoma associated with bilateral ureterohydronephrosis and retroperitoneal perinephric urinoma at the left kidney: A case report and

**DOI:** 10.3892/mi.2025.256

**Published:** 2025-07-28

**Authors:** Anna Thanasa, Efthymia Thanasa, Emmanouil Xydias, Apostolos Ziogas, Ioannis Thanasas

**Affiliations:** 1 Department of Health Sciences, Medical School, Aristotle University of Thessaloniki, 54124 Thessaloniki, Greece; 2 Department of Obstetrics and Gynaecology, EmbryoClinic IVF, 55133 Thessaloniki, Greece; 3 Department of Medicine, University of Thessaly, School of Health Sciences, 41334 Larissa, Greece; 4 Department of Obstetrics and Gynecology, General Hospital of Trikala, 42100 Trikala, Greece

**Keywords:** cervical leiomyoma, ureterohydronephrosis, pelvic urinoma, imaging studies, surgical management, intraoperative complications

## Abstract

The present case report describes the successful surgical treatment of a rare case of a giant cervical leiomyoma of the uterus associated with bilateral ureterohydronephrosis and the formation of a retroperitoneal perinephric urinoma in the left kidney. A 51-year-old patient presented with urinary retention, constipation, and lower abdominal pain radiating mainly to the lumbar region. A clinical examination revealed a small amount of vaginal bleeding, with the uterine fundus palpable ~2 cm below the navel. Imaging with computed tomography and magnetic resonance imaging revealed the presence of a giant cervical leiomyoma of the uterus occupying the entire pelvis, causing compressive effects on the ureters, resulting in bilateral ureterohydronephrosis and the formation of a retroperitoneal urinoma at the level of the left kidney. Tumor markers were negative. It was decided to perform a total abdominal hysterectomy with bilateral salpingo-oophorectomy. The hysterectomy was technically difficult, and the traumatic rupture of the bladder was unavoidable. Surgical drainage of the urinoma was not deemed necessary. The urologists recommended monitoring of the lesion and the post-operative retention of ureteral stents to manage the dilations of the pelvicalyceal system. A histopathological examination of the surgical specimen confirmed the diagnosis of cervical leiomyoma of the uterus. The post-operative course was smooth. Urination was restored without issues following the removal of the urinary catheter. The ureteral stents were removed at 3 months after the surgery, at which time point, the ultrasound examination of the urinary system revealed the complete restoration of the morphology of the kidneys. After the detailed description of this rare case, particular emphasis is placed on the challenging preoperative diagnostic approach and the significant intraoperative difficulties in the effective surgical management of giant cervical leiomyomas of the uterus to ensure the best postoperative outcome.

## Introduction

Leiomyomas, also known as fibroids, are the most common benign gynecological tumors, typically found in premenopausal women. Leiomyomas are diagnosed more frequently in women of African origin compared to Caucasian women (80 vs. 70%); however, the incidence of clinical symptoms in patients of African origin is double than that observed in Caucasian women (1). Despite the high prevalence rates, the pathophysiological mechanisms of uterine leiomyomas remain incompletely understood. Various pathogenic mechanisms have been proposed, involving genes, growth factors, cytokines, chemokines and microRNA deviations (2). In particular, there appears to be a strong association with estrogen and progesterone levels, as the expression of both hormonal receptors is higher in leiomyomas than in the normal myometrium (3). The activation of receptors is hypothesized to lead to the inhibition of key tumor suppression genes, such as p53 and to promote the release of growth factors, ultimately stimulating endometrial development (3). Apart from the hormonal milieu, certain genetic mutations have been found to be associated with the presence of leiomyomas, such as genes located in the 10q24.33 region, which are associated with myogenic differentiation and cytoskeletal structure; impairment of which may explain the proliferation of mesenchymal tissues and the tendency towards the formation of leiomyomas (4). Epigenetic factors have also been examined as potential causes of leiomyoma development, such as microRNAs, which have been observed to be dysregulated in leiomyomas compared to the healthy myometrium and in turn, likely affect gene expression and cellular development, leading to favorable conditions for the formation of fibroids (3,4).

Leiomyomas of the female reproductive system may be located in the uterus or in extrauterine sites (extrauterine leiomyomas). Extrauterine leiomyomas that develop in the broad ligament or, more rarely, in the round ligament, in the uterosacral ligament, or the ovaries are rare (5). From the leiomyomas that develop within the uterus (uterine leiomyomas), those found in the uterine body (corpus uteri) are by far the most common and may be intramural, subserosal or submucosal; while leiomyomas that develop in the cervix of the uterus (cervical leiomyomas) are significantly less frequent (6), being estimated to comprise only a mere 0.6% of all uterine leiomyomas (7).

Cervical leiomyomas, depending on their location within the cervix, can be further classified as extracervical or intracervical leiomyomas, with the latter located within the cervix (as in the case described herein). Extracervical cervical leiomyomas (subserosal), which are characterized by the tumor developing outside the cervical canal, can be further categorized into anterior, posterior and lateral, depending on their position in the cervix. Giant cervical leiomyomas, which can reach a maximum diameter >15 cm, as in the patient in the present study, are extremely rare. Obstructive uropathy accompanied by renal dysfunction is a rare clinical manifestation of these tumors (6).

The present case report describes the successful surgical management of a rare case of giant cervical leiomyoma associated with bilateral ureterohydronephrosis and retroperitoneal perinephric urinoma formation at the level of the left kidney. Furthermore, particular emphasis is placed on the challenging pre-operative diagnostic approach and the significant intraoperative difficulties encountered in the effective surgical management of such complex cases to achieve the best postoperative outcome.

## Case report

A 51-year-old patient, with two previous cesarean sections in her obstetric history, presented to the Emergency Department of the General Hospital of Trikala, Trikala, Greece, complaining of an inability to urinate. Urinary retention was confirmed following the placement of a Foley catheter (no. 18). The patient also reported constipation, dysuria, frequent urination and pain in the lower abdomen extending primarily to the lumbar regions. The onset of symptoms dated back ~6 months, with gradual worsening in intensity. The last normal menstrual period of the patient was 8 months prior. Since then, she had experienced menorrhagia and abnormal vaginal bleeding. Her body mass index was normal (23.9). In her medical history, hypothyroidism was noted, with thyroid hormones well-regulated under medication. There were no reports of recurrent urinary tract infections, chronic kidney disease, or gastrointestinal disorders. According to the patient, this was her first episode of urinary retention.

**Figure 1:**
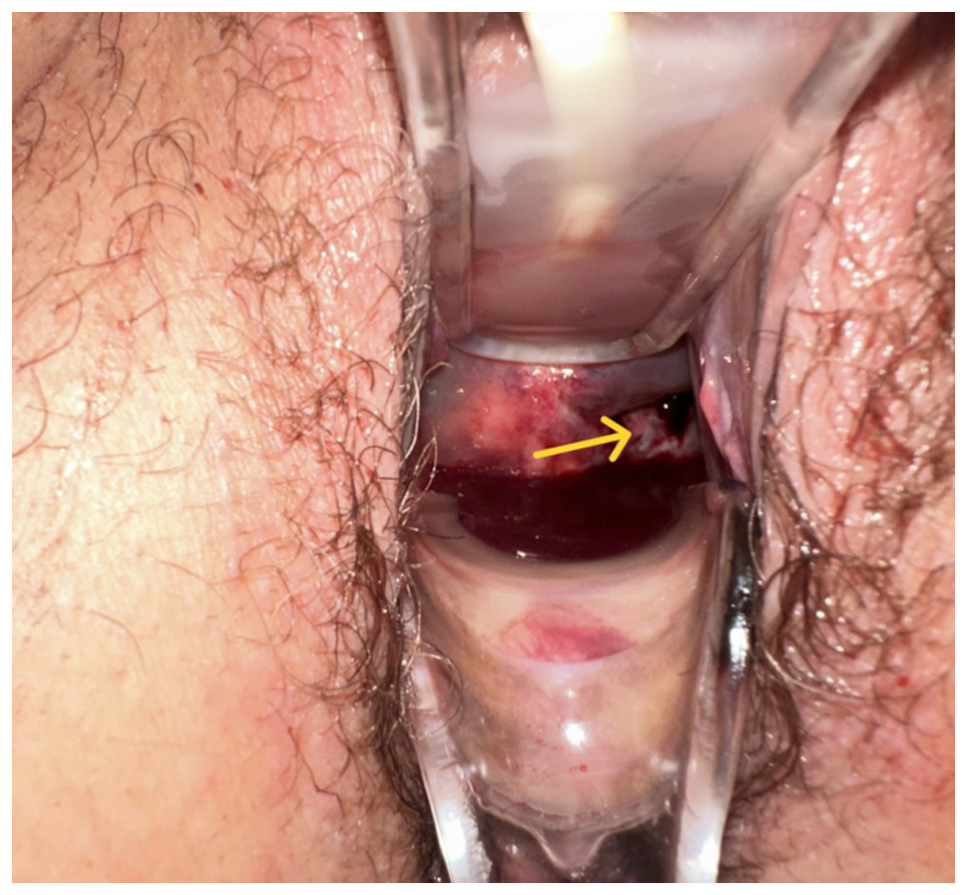
Image obtained during the vaginal examination of the patient, the obliterated and displaced cervix (yellow arrow) supported the presence of a giant endocervical fibroid.

Upon a clinical examination, the abdomen was found to be soft, with no signs of peritoneal irritation. Blood pressure and heart rate were normal (130/80 mmHg and 87 beats/min). The patient's body temperature was 36.8˚C. Tenderness was noted upon the percussion of the kidney areas bilaterally (positive Giordano's sign). Intravenous antibiotic therapy with cefoxitin (Mefoxil^®^) at a dose of 2 g every 8 h was initiated. Of note, ~2 cm below the umbilicus, a hard mass was palpated, suspected to be a uterine fibroid or the uterine fundus. During vaginal examination, minor vaginal bleeding was observed. The cervix was displaced backward and to the left and appeared effaced (Fig. 1). The Pap smear was negative for malignancy. Laboratory tests upon admission revealed mildly elevated inflammation markers. Tumor markers were within normal limits (Table I). Urine culture showed no evidence of urinary tract infection.

**Figure 2:**
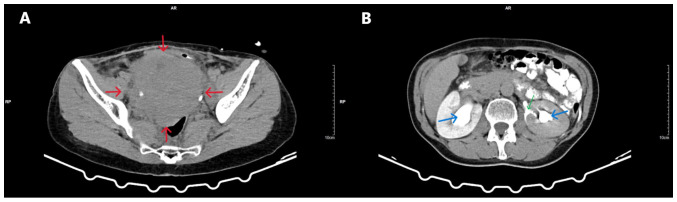
Computed tomography imaging of a giant endocervical fibroid associated with bilateral ureterohydronephrosis and retroperitoneal perinephric urinoma. (A) A large, solid, hyperechoic mass with clear borders (red arrows) occupying the pelvis and compressing adjacent anatomical structures. (B) Bilateral dilation of the pelvicalyceal system (blue arrows) and extravasation of contrast material into the left kidney, corresponding to a retroperitoneal perinephric urinoma (green arrow).

**Figure 3:**
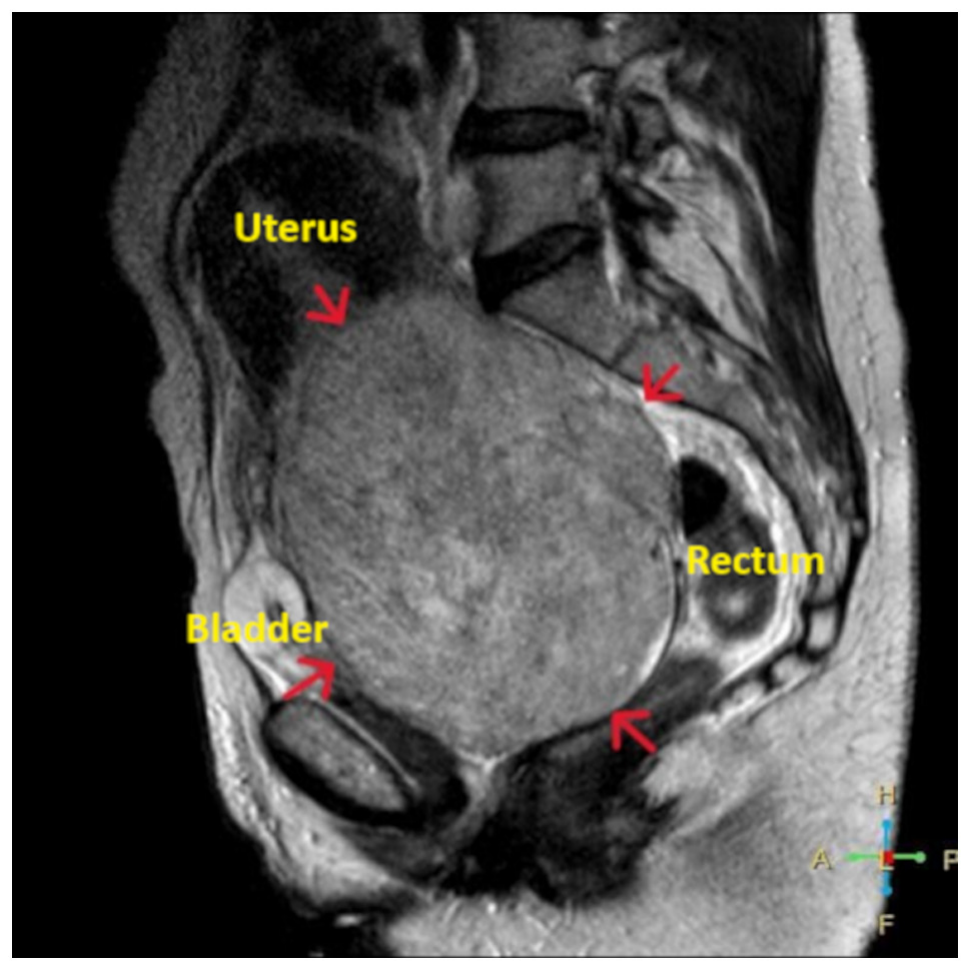
MRI imaging of a well-defined pelvic mass (red arrows) originating from the endocervix corresponding to a giant endocervical fibroid.

Transvaginal ultrasound was non-diagnostic due to the large size of the mass. Similarly, the findings from the transabdominal ultrasound were also non-diagnostic. A computed tomography (CT) scan revealed a large (16x12x10 cm) solid echogenic mass with clear boundaries and signs of cystic degeneration, occupying the entire pelvis, likely originating from the cervix, and causing the compression of the ureters. The non-obstructive dilation of the pelvicalyceal system and the upper and middle third of the right ureter, due to chronic hydronephrotic changes, was observed in the right kidney. In the left kidney, in addition to pelvicalyceal dilation and dilation of the upper and middle third of the left ureter, a small filling defect was noted in the middle calyceal group, with a cup-shaped deformation of the upper and lower calyces and extravasation of contrast into the retroperitoneal space, consistent with a perinephric urinoma (Fig. 2). Magnetic resonance imaging (MRI) revealed a well-defined pelvic mass measuring 16.5x12.5x11 cm, originating from the endocervix. Due to its large size, the mass appeared to displace the endocervix backward and to the left and the uterine body upward, making it palpable through the abdominal wall and suggesting a diagnosis of a giant uterine leiomyoma (Fig. 3). No pathologically enlarged lymph nodes were visible on the current CT or MRI imaging. Pre-operative ureteral stent placement was deemed necessary, both for the intraoperative protection of the ureters and for the therapeutic management of bilateral pelvicalyceal system dilation.

**Figure 4:**
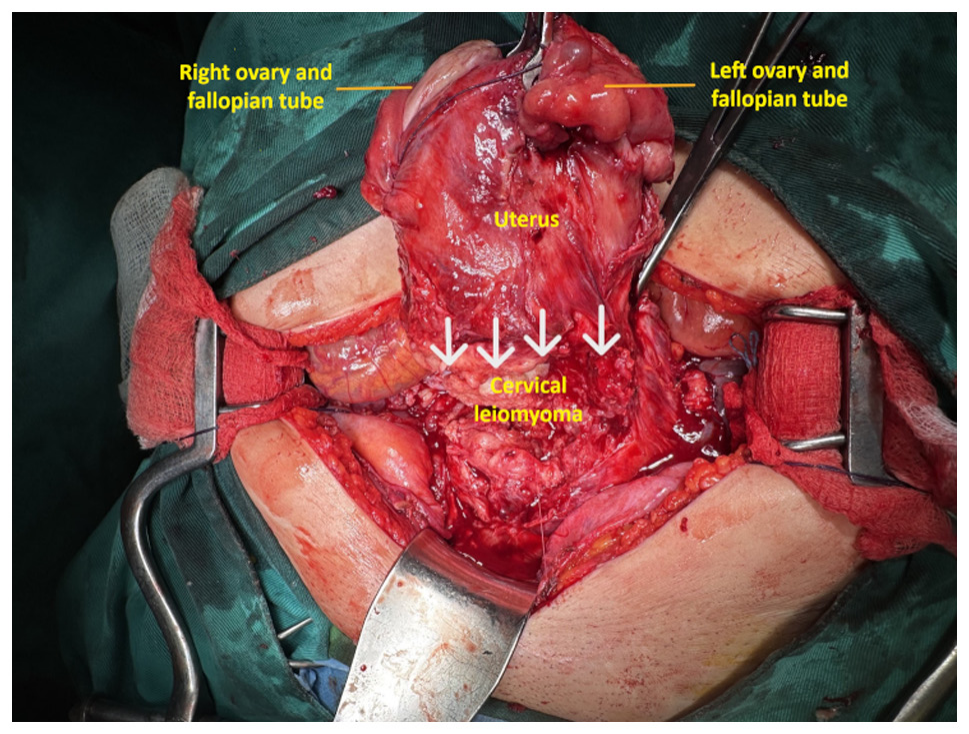
Intraoperative imaging of a giant endocervical fibroid: The presence of the tumor is evident, which is firmly adhered to the adjacent anatomical structures and arises from the isthmus of the uterus at the level of the scars from previous cesarean sections (white arrows), occupying the entire pelvis.

Based on the imaging findings, the diagnosis of giant cervical leiomyoma of the uterus was made. Following the thorough consultation with the surgical team regarding the severity of her condition, the presentation of the available treatment options and after taking into consideration her peri-menopausal status and the absence of further childbearing desire, the patient consented to the recommendation of the team for an abdominal total hysterectomy with bilateral salpingo-oophorectomy. Intraoperatively, a giant cervical leiomyoma was found, wedged in the pelvis and adherent primarily to the anterior pelvic wall due to the previous cesarean sections, making its mobilization difficult. The uterine body and ovaries were not involved in the lesion (Fig. 4). The hysterectomy was technically challenging. The electrothermal bipolar vessel sealing device (LigaSure^™^) significantly aided in reducing the risk of intraoperative bleeding. For the intraoperative blood loss and hemodynamic stabilization of the patient, a total of 3 units of whole blood and 1 unit of plasma were transfused (2 units of blood and 1 plasma intraoperatively, and 1 unit of blood on the second postoperative day) (Table I). During surgical maneuvers, a traumatic rupture of the bladder ~5 cm in length occurred, which was repaired in layers. Urological intervention was not required to drain the urinoma. It was deemed appropriate to monitor the lesion and leave the ureteral stents in place post-operatively to manage the dilation of the pelvicalyceal system.

**Figure 5:**
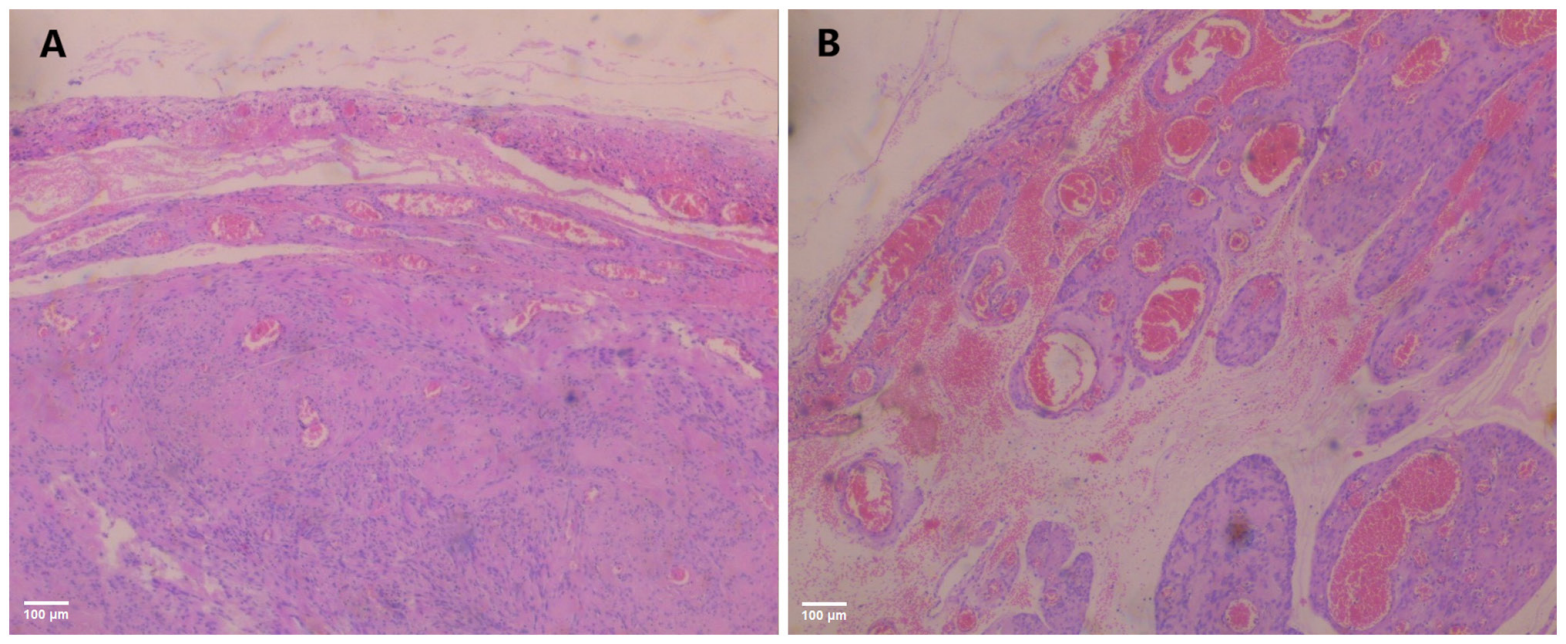
Histological image of a cervical fibroid. (A) Depiction of smooth borders with the absence of mitosis and atypia (hematoxylin and eosin staining; magnification, x40; scale bar, 100 µm). (B) Vascular congestion with focal hemorrhagic infiltration of the stroma (hematoxylin and eosin staining; magnification, x40; scale bar, 100 µm).

A histological examination of the surgical specimen was performed in accordance with routine protocols of the authors' laboratory. Specimens were embedded in paraffin cubes and sections with a thickness of 5 µm were obtained for analysis. A buffered, 10% formalin solution was utilized as a fixative medium, for 36 h at room temperature. Hematoxylin and eosin 0.5% alcohol solution (Diachel A.E.) staining was used, at room temperature with a 12-min duration. All microscopic examinations were performed using a LEICA DM2000 optical microscope (Leica Microsystems GmbH). The histological examination of the surgical specimen confirmed the diagnosis of cervical leiomyoma of the uterus. A microscopic examination revealed mild to moderate cellularity with no mitoses. Necrosis or cytological atypia was not observed (Fig. 5).

**Figure 6:**
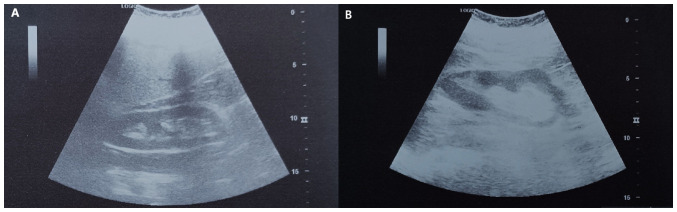
Post-operative ultrasound examination of the urinary system, revealing normal kidney morphology without the pre-operative dilatation of the pyelocaliceal system and the presence of the perinephric urinoma; (A) right kidney, (B) left kidney.

Following an uneventful post-operative course, the patient was discharged on the 5th post-operative day. The urinary catheter was removed at 5 days after discharge. Urinary function was restored without issues. Following the recommendation of the urologists, the ureteral stents were removed 3 months after surgery, at which time the renal ultrasound was normal, without pelvicalyceal system dilation or perinephric urinoma (Fig. 6).

## Discussion

The clinical diagnosis of giant cervical leiomyomas of the uterus presents a challenge in everyday medical practice. The clinical symptoms are non-specific and usually relate to the pressure of the enlarged cervical leiomyoma on adjacent pelvic organs, occupying the entire pelvic cavity. Therefore, pain localized in the lower abdomen, primarily radiating to the lumbar regions or, less frequently, to the kidney areas, abdominal distension, intense dysuric discomfort, urinary retention and constipation are the most common clinical manifestations of giant cervical leiomyomas of the uterus (8). In contrast to uterine body fibroids, abnormal uterine bleeding is not a frequent symptom in patients with cervical leiomyomas (9). Thus, it is not surprising that the patient described herein did not report symptoms related to menstruation. In addition, it is not unexpected that constipation, urinary retention and hydronephrosis were the main symptoms in the patient described herein. A potential surprise could be the non-traumatic and non-obstructive rupture of the renal parenchyma and the formation of a retroperitoneal perinephric urinoma, a clinical entity that is uncommon in practice.

An urinoma is an encapsulated collection of extravasated urine leaking from the urinary system and accumulating in a cavity surrounded by a fibrous capsule in the perinephric or periureteral space. This rare clinical entity, the severity of which varies from asymptomatic cases (small retroperitoneal urinomas after extraperitoneal urine leakage) to causing acute abdomen with signs of peritonitis in cases where the urine leakage is intraperitoneal, may be caused by trauma, surgery, or urinary tract obstruction (10). Urolithiasis, retroperitoneal fibrosis and the presence of a large intra-abdominal mass primarily located in the pelvis are key causes of urinoma formation, which is not due to iatrogenic surgical trauma (11,12). In the patient in the present study, the pressure from the giant cervical leiomyoma on the ureters resulted in impaired urine flow, causing non-traumatic rupture of the renal parenchyma and the formation of a retroperitoneal urinoma in the left kidney. The early diagnosis of the urinoma, usually confirmed by a CT scan or MRI, and prompt, appropriate conservative or surgical management depending on the case is critical for avoiding complications, such as infection leading to the formation of perinephric or periureteral abscesses and transient or permanent renal damage (13). In the patient in the present study, due to the small size of the urinoma and the absence of acute symptoms, urologists did not recommend surgical intervention for drainage. It was deemed appropriate to monitor the lesion and leave the ureteral stents in place postoperatively to address the dilatation of the pelvicalyceal system.

The imaging of large cervical leiomyomas of the uterus is crucial for an accurate pre-operative diagnostic approach to these tumors originating from the cervix, treatment planning and minimization of surgical complications. Ultrasound, CT scan and MRI play a crucial role in the management of patients with cervical leiomyomas (14). Ultrasound is the most frequently used imaging method. With both transabdominal and transvaginal ultrasound, a well-defined hypoechoic mass can be observed in the endocervix or outside the cervical canal, which typically contains solid components, as well as areas of cystic necrosis, internal vascularization and calcification. Ultrasound findings compatible with the presence of mixed echogenic tumors with central necrosis and irregular vascular distribution are observed in ~20% of large cervical leiomyomas, which require differential diagnosis from malignant cervical lesions (15). In cases with a strong suspicion of malignancy in the cervix, a CT scan or MRI are performed for the further imaging evaluation of the cervical mass. In an MRI, which provides better visualization of the lateral and posterior pelvic regions, leiomyomas appear as sharply demarcated areas of low to intermediate signal intensity on both T1 and T2 sequences (16). Although an MRI cannot confirm the diagnosis of malignant cervical lesions, compared to an ultrasound, it has higher specificity and positive predictive value (17). In the patient described herein, due to the large size of the tumor, neither transvaginal nor transabdominal ultrasound was diagnostic. An urgent CT scan was performed, which established the diagnosis of cervical leiomyoma and associated urinary tract lesions (hydronephrosis, urinoma). The scheduled MRI was deemed necessary for better delineating the specific imaging characteristics of the cervical mass and its anatomical relationship with pelvic structures to optimize surgical planning.

The presence of large pelvic masses, particularly in the narrow pelvis minor area, such as in the present case, can severely distort normal, expected anatomy. The use of pre-operative imaging modalities in order to accurately explore and map out these distortions can significantly assist intra-operatively, as the surgeon is better prepared to work in the distorted surgical site, is better aware of the size, location and distance of the tumor relative to other organs, and is thus less likely to cause injury and other complications (18). While an ultrasound is a readily available and dynamic test, its subjective nature and inability to adequately visualize large tumors, such as in the present case, render it of limited value in the context of pre-operative preparation for advanced cases (18). Tomographic methods, such as CT scans and particularly, MRI, constitute the gold standard for visualizing the entire pelvis and for facilitating identification of the tumor, its location, size and association with healthy tissue; these methods are therefore necessary to better plan the surgery and reduce the risk of complications (19). This was also the case for the patient described herein, where both techniques were performed and MRI was primarily used to plan the surgery. However, the possibility remains that despite careful preparation and planning, complications may occur regardless, in which case the surgeon needs to be able to effectively address them. This was true in the present case as well, where despite careful preparation and planning, a rupture of the urinary bladder occurred during surgical maneuvering due to the size of the primary tumor and the presence of multiple adhesions in the pelvic cavity; however, it was promptly and effectively addressed and repaired without long term effects on the patient's health and quality of life.

The surgical management of giant cervical leiomyomas of the uterus is challenging and is largely dependent upon the condition and wishes of the patient. With the primary complaint among most gynecological patients being the provision of lacking information regarding their surgery and the lack of opportunities to influence the selection of their treatment; thorough consultation with the gynecological surgery team regarding the underlying condition, its severity, the available treatment options and the advantages and disadvantages of each one is of paramount importance (20). It is vital that the surgical team presents and analyzes all available treatment options, even those that go against their recommendations and clarifies what precise intervention they propose in addition to the reason or indication why it is proposed over the other alternatives (20). The matter of surgical route should also be discussed, along with the advantages and disadvantages of each route, in addition to issues of treatment availability at the center of care (20). Finally, this information should ideally be discussed well in advance of the arranged date of surgery at the outpatient office, in order to ensure adequate time and a stress-free environment for the patient to participate in the decision-making process and to provide informed consent to a mutually agreeable treatment option (20).

Hysterectomy or myomectomy, depending on the age and desire of the patient for future pregnancy, are recommended as the main therapeutic options for patients with large cervical leiomyomas (7). Ideally, minimally invasive surgery should be preferred over laparotomy, since it is associated with reduced patient morbidity rates. However, in the current case, the presence of large tumors combined with the presence of severe peritoneal adhesions renders the laparoscopic route extremely challenging and thus laparotomy may be preferred (21). In patients who are an increased surgical risk, where the selection of a minimally invasive approach is more strongly recommended, robotic surgery may be selected, as it combines the efficacy of laparotomy with the reduced morbidity of laparoscopy (22); however, this option is highly dependent upon availability. Abdominal cervicectomy for the management of a giant cervical leiomyoma is extremely difficult and may be attempted in cases where the goal is to preserve the fertility of the patient (15). Since the patient in the present case report did not wish to pursue such an option, this method was rejected; however, it may be a viable option for younger patients with an incomplete family plan (23).

A treatment alternative, apart from surgical excision is arterial embolization. Most commonly performed for leiomyomas in the uterine body, uterine artery embolization provides a minimally invasive treatment option where blood supply to the leiomyoma is blocked, resulting in tumor shrinkage and symptom improvement, with the preservation of the uterus. While this methodology is not commonly applied to cervical leiomyomas, due to the complexity and variation of cervical vessels, specialized centers have managed to apply this technique to a limited amount of patients with favorable outcomes, both in terms of surgical morbidity and an improvement in quality of life (24). Recently, an interesting variation of this technique pertaining to a case of successful treatment of a large symptomatic cervical leiomyoma with bilateral ovarian artery embolization without embolization of the uterine artery was described in the literature (25). Although it pertains to a single patient case, under conditions where the potential risks regarding fertility and hormonal function are managed, this method could be considered a viable alternative in the treatment of symptomatic cervical leiomyomas, protecting patients from a potentially complicated and extremely hemorrhagic surgical procedure (25). Definitive conclusions regarding the optimal treatment option for cervical leiomyomas are difficult to make, given their rarity compared to other leiomyoma types. However, in the most recent meta-analysis on the topic by Ferrari *et al* (26) the vast majority of the patients (88%) were treated with surgery, compared to only 10% by arterial embolization, indicating a clear preference towards the former. Furthermore, embolization treatments were successful only in 55.5% of cases, indicating lower efficacy than traditional surgery; however, surgery was associated with 5.6% total complication rate, indicating relatively lower safety (26). The authors of that study ultimately stress the challenging nature of cervical myoma treatment regardless of the approach used and recommend that these procedures are only undertaken by experienced teams, with embolization treatments being promising, but with limited available data (26).

The increased risk of intraoperative bleeding and the potential for inadvertent trauma to adjacent organs compressed by the giant tumor (bladder, ureters and rectum) render the surgical management of these patients extremely difficult (7). In the patient in the present study, who did not wish to pursue future pregnancy, abdominal total hysterectomy with bilateral salpingectomy and oophorectomy was selected. The surgery presented significant difficulties. Multiple and firm adhesions between the cervical leiomyoma and the bladder and anterior abdominal wall, due to previous cesarean sections, combined with the displacement of these structures by the massive size of the tumor, made mobilization of the uterus and cervical leiomyoma in the surgical field challenging (Fig. 4). This led to significant intraoperative bleeding, a traumatic rupture of the bladder wall, and an increased risk of ureteral injury. To reduce the risk of intraoperative bleeding, the use of the electrothermal bipolar vessel sealing device (LigaSure^™^) was considered crucial. The bladder wall rupture was sutured, and the integrity of the ureters was checked. Pre-operative cystoscopy and bilateral ureteral stent placement allowed intraoperative tracing of the ureters and prevented inadvertent injury (7). Additionally, proper and complete preoperative planning, well-organized operating room settings, and the sufficient experience and skill of the surgical team with a thorough knowledge of pelvic anatomy, which is often distorted by the giant cervical leiomyoma, are essential for the successful outcome of the surgical procedure (27).

In conclusion, giant cervical uterine fibroids are extremely rare. Transient, non-traumatic or non-obstructive lesions of the ureters and kidneys related to the pressure exerted by the giant cervical fibroid may occur. The diagnostic and therapeutic approach to these fibroids is challenging. An additional challenge, which would make the management of our patient even more difficult, would be her desire to preserve her uterus and fertility. However, regardless of the decision to perform a hysterectomy or myomectomy, managing these patients remains difficult even for experienced gynecologists and depends largely on the individual circumstances of the patient, the organization of the medical center, and the coordination of the surgical team.

## Figures and Tables

**Table I:** Laboratory tests of the patient from the day of admission to the clinic until the day of discharge.

Laboratory tests	Day of admission to the clinic	Day before surgery	1st post-operative day	2nd post-operative day	5th post-operative day	Normal laboratory values
Ht	36.24%	33.85%	26.1%	23.4%	28.2%	37.7-49.7%
Hb	12.1 gr/dl	11.6 gr/dl	8.5 gr/dl	7.5 gr/dl	8.9 gr/dl	11.8-17.8 gr/dl
PLT	275x10^3^/ml	271x10^3^/ml	165x10^3^/ml	158x10^3^/ml	194x10^3^/ml	150-350 x10^3^/ml
WBC	11.3x10^3^/ml	9.2x10^3^/ml	21.7x10^3^/ml	15.3x10^3^/ml	9.1x10^3^/ml	4-10.8 x10^3^/ml
NEUT	78.9%	71.1%	91.5%	81.4%	69.3%	40-75%
CRP	0.75 mg/dl	0.45 mg/dl				<0.7 mg/dl
APTT	29.8 sec	28.6 sec	34.1 sec	36.1 sec	31.1 sec	24.0-35.0 sec
INR	0.92	0.90	1.02	1.07	0.97	0.8-1.2
FIB	255 mg/dl	251 mg/dl	222 mg/dl	215 mg/dl	227 mg/dl	200-400 mg/dl
Glu	110 mg/dl	85 mg/dl	91 mg/dl	87 mg/dl	85 mg/dl	75-115 mg/dl
Cr	0.75 mg/dl	0.63 mg/dl	0.67 mg/dl	0.61 mg/dl	0.62 mg/dl	0.40-1.10 mg/dl
Κ^+^	3.7 mmol/l	3.9 mmol/l	3.4 mmol/l	4.2 mmol/l	3.9 mmol/l	3.5-5.1 mmol/l
Να^+^	141.4 mmol/l	142.3 mmol/l	137.3 mmol/l	139.4 mmol/l	141.1 mmol/l	136-145 mmol/l
B	0.48 mg/dl	-	-	-	0.74 mg/dl	0.3-1.2 mg/dl
SGOT	29 IU/l	-	-	-	27 IU/l	5-33 IU/l
SGPT	27 IU/l	-	-	-	24 IU/l	10-37 IU/l
CEA	2.85 ng/ml	-	-	-	-	<5 ng/ml
CA 125	18.3 U/ml	-	-	-	-	≤35 U/ml
CA 15-3	19.4 U/ml	-	-	-	-	0.0-31.3 U/ml
CA 19-9	13.1 U/ml	-	-	-	-	0.0-37 U/ml

Ht, hematocrit; Hb, hemoglobin; PLT, platelets; WBC, white blood cells; NEUT, neutrophils; CRP, C-reactive protein; APTT, activated partial thromboplastin time; INR, international normalized ratio; FIB, fibrinogen; Glu, glucose; Cr, creatinine; K^+^, potassium; Na^+^, sodium; B, bilirubin; SGOT, serum glutamic oxaloacetic transaminase; SGPT, serum glutamate pyruvate transaminase; CEA, carcinoembryonic antigen; CA125, cancer antigen 125; CA15-3, cancer antigen 15-3; CA15-9, cancer antigen 19-9.

## Data Availability

The data generated in the present study may be requested from the corresponding author.
